# Fenton Catalytic Degradation of Rhodamine B by Zero-Valent Iron/Alumina Catalyst

**DOI:** 10.3390/molecules31132270

**Published:** 2026-06-29

**Authors:** Kexin Ge, Shuaiqi Chen, Boning Jiang, Xuhui Wang, Xiangyu Xu, Jiaqing Song

**Affiliations:** 1 College of Chemistry, Beijing University of Chemical Technology, Beijing 100029, China; 2024210791@mail.buct.edu.cn (K.G.); 2022430033@mail.buct.edu.cn (S.C.); 2023400285@mail.buct.edu.cn (B.J.); songjq@mail.buct.edu.cn (J.S.); 2 State Key Laboratory of Chemical Resource Engineering, Beijing University of Chemical Technology, Beijing 100029, China; 3 Sinopec Catalyst Company Limited, Beijing 100176, China; xuhuiwang@126.com

**Keywords:** zero-valent iron, alumina, heterogeneous Fenton, Rhodamine B, hydroxyl radical, catalytic degradation

## Abstract

Rhodamine B (RhB) is a typical xanthene-based cationic dye. Its widespread application has brought serious safety and environmental risks. Heterogeneous Fenton systems based on zero-valent iron (Fe^0^) are promising for RhB degradation. However, bare Fe^0^ suffers from severe agglomeration and surface passivation. In this study, alumina with a large pore volume and high specific surface area was employed as a support to enhance Fe^0^ dispersion and stability. The catalyst was prepared via a glucose-assisted carbothermal reduction method, and the formation of Fe^0^ was confirmed by X-ray diffraction and electron microscopy analyses. Under optimal conditions (pH = 3.58, catalyst dosage = 0.8 g·L^−1^, H_2_O_2_ = 10 mM), 10 mg·L^−1^ RhB was completely degraded within 25 min, with a pseudo-first-order rate constant of 0.432 min^−1^. This exhibits a faster degradation rate and efficiency advantage. Radical quenching experiments indicated that hydroxyl radicals (•OH) were the dominant reactive species, while singlet oxygen (^1^O_2_) also contributed to the degradation process. Two primary degradation pathways, including N-deethylation and hydroxylation, were identified. The catalyst showed moderate reusability with slight deactivation after repeated cycles. This study demonstrates that tailoring the pore structure of alumina supports is an effective strategy to enhance Fe^0^ dispersion, mass transfer, and catalytic performance in heterogeneous Fenton systems.

## 1. Introduction

The discharge of toxic organic dyes into aquatic ecosystems poses a serious threat to ecological and human health [1]. Among these pollutants, RhB is a representative cationic dye characterized by a stable molecular structure, strong chromophoric groups, and poor biodegradability [2,3]. Owing to its extensive use in textile, printing, and plastic industries, efficient removal of RhB from wastewater remains a critical challenge [4,5,6]. Advanced oxidation processes (AOPs) have been widely explored for the degradation of refractory organic pollutants [7,8,9,10,11,12,13,14,15]. Among them, the Fenton process is particularly attractive due to its simplicity and high oxidation efficiency [16]. Compared with homogeneous Fenton systems, heterogeneous Fenton catalysts offer advantages including less iron sludge production, easier solid–liquid separation, and better reusability [17]. Among these, iron-based heterogeneous catalysts have become a research hotspot in this field owing to their low cost, non-toxicity, and environmental friendliness characteristics. Iron-based heterogeneous catalysts mainly include Fe^0^ [18,19], iron oxides [20,21], and multimetallic catalysts [22,23]. Fe^0^ is considered highly effective due to its ability to continuously generate Fe^2+^ for H_2_O_2_ activation. However, bare Fe^0^ nanoparticles tend to aggregate and undergo surface passivation, leading to decreased active site availability and limited catalytic performance [24].

In recent years, many researchers have conducted degradation experiments on RhB. As shown in Table 1, significant differences exist among various systems in terms of catalytic activity, applicable pH range, hydrogen peroxide utilization efficiency, and mineralization capability. Meanwhile, it can be found from these studies that the support structure plays a crucial role in Fenton-like reactions, and specific surface area and pore structure cannot simply substitute for each other. Therefore, the generally low degradation efficiency in Table 1 may be due to the nature of the material itself, the small specific surface area of the support, or an insufficient amount of active components. Zhou et al. established a linear relationship between the apparent rate constant and the surface area concentration (SA/V), demonstrating that an increase in surface area can directly enhance the surface decolorization reaction rate [25]. This also indicates that the specific surface area mainly determines the dispersion degree of active components, the number of active sites that can be loaded per unit mass of support, and the density of surface reaction sites. On the other hand, pore volume, pore size, and pore connectivity more directly influence the diffusive mass transfer of RhB and hydrogen peroxide to the active sites. Especially for organic dyes with relatively large molecular sizes such as Rhodamine B, mesopores and larger pore volumes are more favorable for reducing internal diffusion resistance, improving site accessibility, and alleviating pore blockage after loading. Peng et al. emphasized that the development of high-performance catalysts largely depends on improving the accessibility and utilization efficiency of active sites through rational pore structure engineering [26]. A high specific surface area can provide more exposed active sites for Fe^0^ dispersion, whereas a large pore volume and interconnected pore channels facilitate the diffusion and transport of reactants and intermediates within the catalyst framework. Therefore, a support simultaneously possessing a large pore volume and high specific surface area is expected to effectively alleviate the mass-transfer limitations and aggregation problems commonly encountered in Fe^0^-based heterogeneous Fenton systems.

Accordingly, alumina with a large pore volume and high specific surface area was employed as the support to construct a zero-valent iron/alumina heterogeneous Fenton catalyst. Fe^0^ was introduced through a glucose-assisted carbothermal reduction strategy to improve the dispersion of active species and enhance catalytic performance toward RhB degradation [27]. The effects of iron loading, support pore structure, initial RhB concentration, solution pH, catalyst dosage, and H_2_O_2_ dosage on catalytic degradation performance were systematically investigated. In addition, the reactive species, degradation pathways, and catalyst reusability were analyzed to clarify the catalytic mechanism of the system. This work provides a deeper understanding of the structure–activity relationship between pore structure and heterogeneous Fenton catalytic performance, and offers a feasible strategy for the design of efficient iron-based catalysts for organic dye wastewater treatment.

**Table 1:** Comparison of Rhodamine B treatment performance among different Fenton-like systems.

Catalysts	Reaction Conditions	Catalytic Performance	References
Cu/Al_2_O_3_/g-C_3_N_4_	pH: 4.9; C_0_: 20 mg∙L^−1^; H_2_O_2_: 10 mM; dosage: 1 g∙L^−1^	removal rate: 96.4% k: 6.47 × 10^−4^ s^−1^	[25]
Fe^0^/H_2_O_2_	pH: 4.0; C_0_: 0.1 mM; H_2_O_2_: 8 mM; dosage: 9 mM	removal rate: 98%	[28]
Cu(II)-pyridyl/silica microspheres	pH: 7.1; C_0_: 5 mg∙L^−1^; H_2_O_2_: 300 mg∙L^−1^; dosage: 2 g∙L^−1^	removal rate: 96%; k: 0.665 h^−1^	[29]
HPS-0.15LFO (photo-Fenton)	pH: 6.0; C_0_: 10 mg∙L^−1^; H_2_O_2_: 15 mM; dosage: 1 g∙L^−1^	removal rate: 98.9%; k: 0.0544 min^−1^	[30]
K-Fe (photo-Fenton)	pH: 7.2; C_0_: 10 mg∙L^−1^; H_2_O_2_: 0.5 mM; dosage: 1 g∙L^−1^	removal rate: 98.8%; k: 0.3155 min^−1^	[31]
AC@Fe/3	pH:4.2; C_0_:50 mg∙L^−1^; H_2_O_2_: 8 mM; dosage: 0.27 g∙L^−1^	removal rate: 96.1%; k: 0.006 min^−1^	[32]
Fe/MCM-41	pH:4; C_0_:100 mg∙L^−1^; H_2_O_2_: 20 mM; dosage: 1 g∙L^−1^	removal rate: 92.4% k: 0.164 min^−1^	[33]

## 2. Results and Discussion

### 2.1. Material Characterization

**Figure 1:**
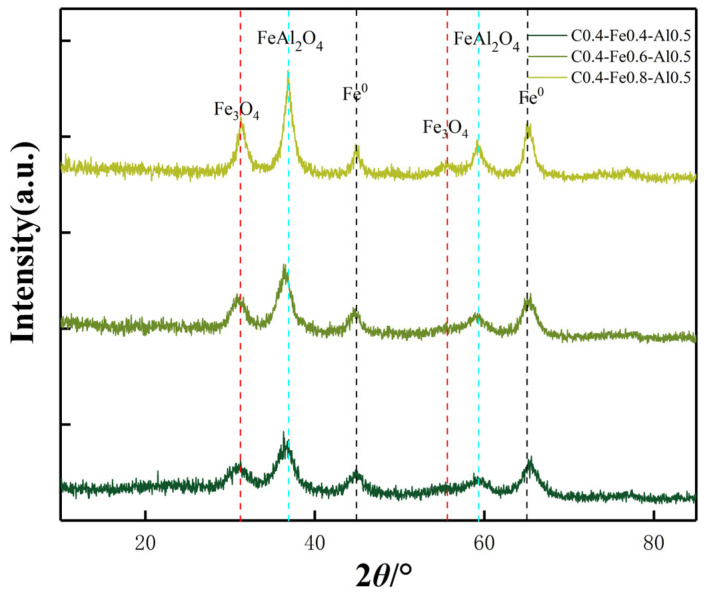
XRD of zero-valent iron/alumina catalysts with different ferric chloride impregnation amounts.

As shown in Figure 1, the characteristic diffraction peaks at 2θ = 44.7° and 65.1° correspond to the (110) and (200) crystal planes of zero-valent iron (Fe^0^, PDF: 01-089-7194), confirming the successful formation of Fe^0^. Peaks at 36.9° and 59.3° are assigned to FeAl_2_O_4_ (PDF: 01-089-1679). The peak at 31.2° corresponds to Fe_3_O_4_ (PDF: 01-076-0957). With increasing ferric chloride impregnation amount, the diffraction peaks associated with Fe^0^ and iron-containing phases gradually intensified, indicating an increase in the content and crystallinity of iron-related species within the catalysts. Notably, the C0.4-Fe0.8-Al0.5 sample exhibited a distinct diffraction peak at approximately 2θ = 55.6°, corresponding to Fe_3_O_4_. This result suggesting that excessive iron loading may lead to incomplete reduction of iron species or partial oxidation of Fe^0^ during cooling and air exposure. Therefore, the iron impregnation amount not only influences the generation of Fe^0^ but also affects the relative proportions of Fe^0^, iron oxides, and FeAl_2_O_4_ spinel phases. Similar phase transformation behavior has also been reported in previous studies on Fe-containing alumina systems [34].

**Figure 2:**
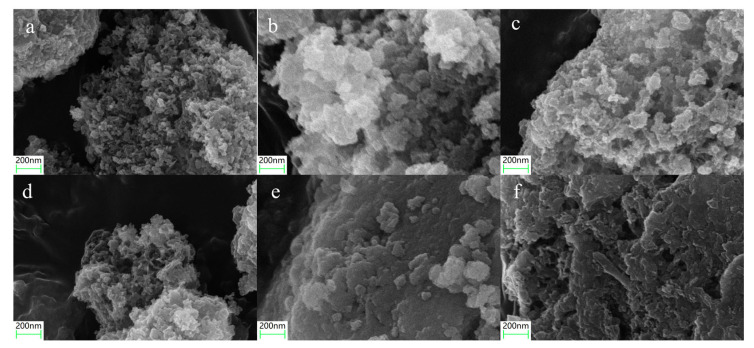
SEM images of the zero-valent iron/alumina catalysts with different ferric chloride impregnation amounts ((**a**) C0.4-Fe0.4-Al0.5; (**b**) C0.4-Fe0.5-Al0.5; (**c**,**d**) C0.4-Fe0.6-Al0.5; (**e**) C0.4-Fe0.7-Al0.5; (**f**) C0.4-Fe0.8-Al0.5).

As shown in Figure 2, the iron impregnation amount has a significant influence on the pore structure of the catalysts: At relatively low iron loading, the catalyst surface still maintained an obvious porous structure. However, as the ferric chloride impregnation amount increased, the pore channels gradually became filled by iron-containing species. In particular, the internal pore structure of C0.4-Fe0.7-Al0.5 and C0.4-Fe0.8-Al0.5 nearly disappeared, indicating severe pore blockage and structural coverage caused by excessive iron deposition. Table 2 summarizes the BET surface area and pore volume of Fe^0^/Al_2_O_3_ catalysts prepared using different alumina supports and FeCl_3_ loadings. The specific surface area of C0.4-Fe0.4-Al0.5 is 208.1 m^2^/g, the pore volume is 0.28 cm^3^/g, and the average pore size is 5.38 nm. As the Fe loading increased, the specific surface area and pore volume decline, and the average pore size increases from 5.38 nm to 7.59 nm.

This trend suggests that the textural properties of the catalysts were primarily governed by those of the alumina supports. The decline in specific surface area and pore volume at higher Fe loadings can be attributed to the deposition of iron species within the pore channels and on the support surface, leading to partial pore blockage and reduced pore accessibility.

After calcining the support at 1000 °C, its dry basis content can be calculated to be 91%. Combined with the known Si/Al and P/Al molar ratios, the theoretical *Fe/Al* molar ratio in C0.4-FeZ-Al0.5 can be calculated according to Equation (1):

(1) $$Fe/Al=\frac{Z/270.3}{0.5*0.91/(102/2+x_{1}*60/2+x_{2}*142/2)}$$

where x_1_ represents the Si/Al molar ratio corresponding to the actual amount of silicon incorporated; x_2_ represents the P/Al molar ratio corresponding to the actual amount of phosphorus incorporated; and Z is 0.6.

**Figure 3:**
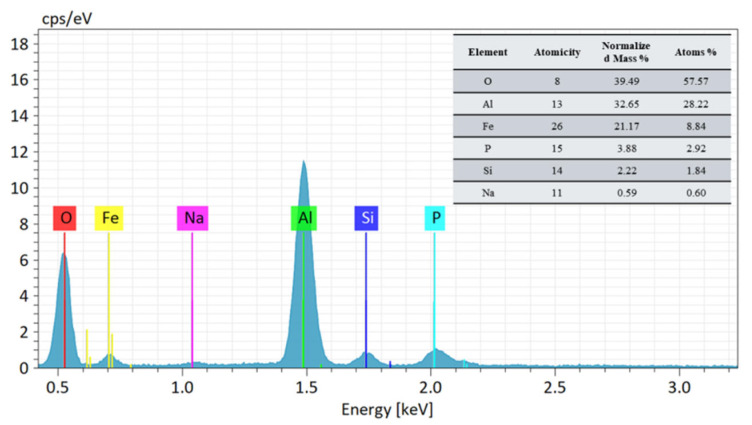
EDS spectrum of C0.4-Fe0.6-Al0.5.

To further evaluate the actual iron loading, the *Fe/Al* molar ratio of C0.4-Fe0.6-Al0.5 was analyzed by EDS. Figure 3 shows the EDS spectrum of C0.4-Fe0.6-Al0.5. The experimentally determined *Fe/Al* molar ratio was 0.313, which is close to the theoretical value of 0.294, demonstrating that the impregnation strategy based on FeCl_3_·6H_2_O effectively introduced iron species onto the alumina support.

**Figure 4:**
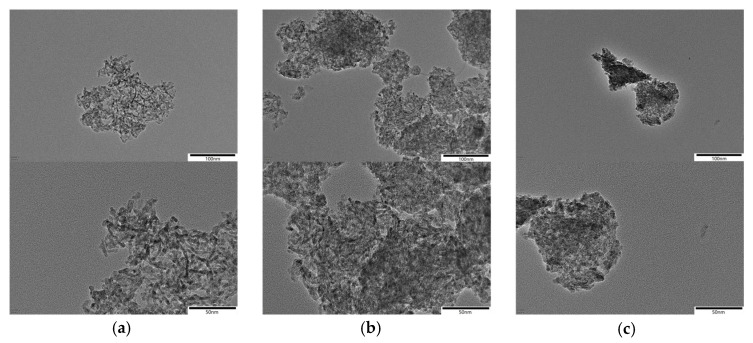
TEM images of zero-valent iron/alumina catalysts with different ferric chloride impregnation amounts ((**a**) C0.4-Fe0.4-Al0.5; (**b**) C0.4-Fe0.6-Al0.5; (**c**) C0.4-Fe0.8-Al0.5).

The TEM images shown in Figure 4 further confirm the influence of iron loading on the pore structure and morphology of the catalysts. C0.4-Fe0.4-Al0.5 still exhibited a relatively loose and interconnected pore structure. Although partial pore filling occurred in C0.4-Fe0.6-Al0.5, a certain degree of pore structure was still preserved. In contrast, the surface of C0.4-Fe0.8-Al0.5 was heavily covered by iron-containing particles, making the original pore structure difficult to distinguish.

**Figure 5:**
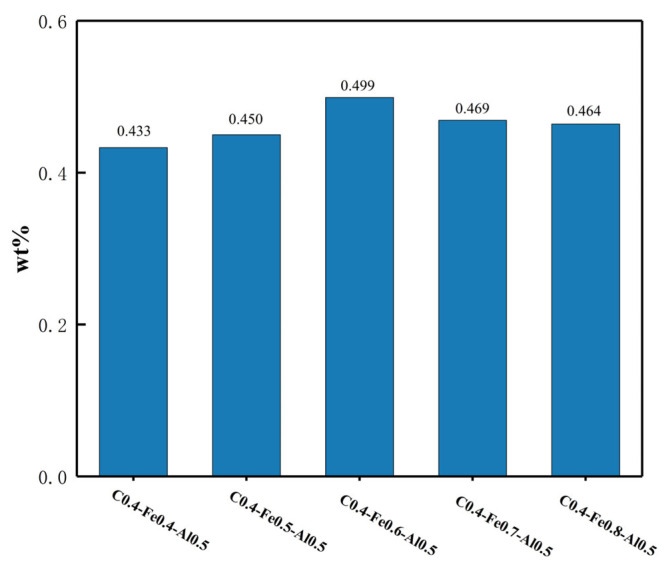
Fe^0^ content of zero-valent iron/alumina catalysts with different ferric chloride impregnation amounts.

To quantitatively determine the Fe^0^ content in the catalysts, Fe^0^ titration was performed, and the results are shown in Figure 5. The Fe^0^ content initially increased and subsequently decreased with increasing ferric chloride impregnation amount. Combined with the synergistic analysis in Figure 1, the interfacial reaction and phase transformation mechanisms at different iron loading stages can be elucidated. At relatively low iron loading, part of the iron reacted with the alumina support to form FeAl_2_O_4_ spinel, resulting in a relatively low proportion of reducible Fe species and consequently low Fe^0^ content [34]. As the iron loading increased, more iron species were reduced to Fe^0^, leading to a significant increase in Fe^0^ content. However, when the iron loading further exceeded the optimal level, excessive Fe^3+^ not only promoted the formation of FeAl_2_O_4_ spinel through interfacial reactions with alumina [35], but also accelerated the oxidation and consumption of Fe^0^. Consequently, the Fe^0^ content decreased significantly at high iron loading. These results suggest that the catalytic performance of the system is closely associated with the balance among Fe^0^ generation, pore structure preservation, and phase transformation behavior.

### 2.2. Catalytic Performance

#### 2.2.1. Effect of Iron Loadings

**Figure 6:**
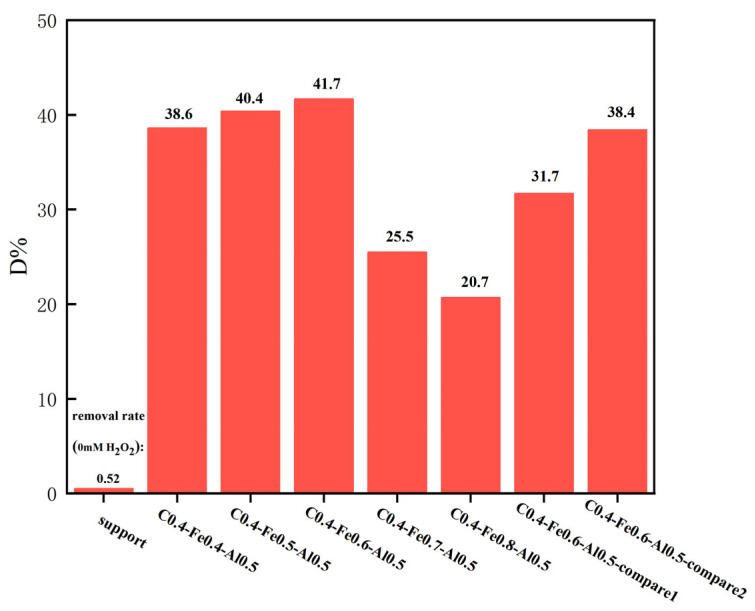
Degradation efficiency of RhB over catalysts with different ferric chloride impregnation amounts.

To investigate the influence of Fe^0^ content on heterogeneous Fenton catalytic performance, catalysts with different ferric chloride impregnation amounts were prepared and evaluated for RhB degradation. As summarized in Table 3 and Figure 6, the degradation efficiency of RhB initially increased and subsequently decreased with increasing iron loading. C0.4-Fe0.6-Al0.5 shows the highest efficiency (41.7%), owing to sufficient Fe^0^ active sites and well-preserved pore structure. This enhancement can be mainly attributed to the increased Fe^0^ content and improved availability of catalytic active sites, which promoted H_2_O_2_ activation and reactive oxygen species generation. Excessive iron loading causes particle aggregation, pore blockage, and reduced Fe^0^ proportion, leading to decreased performance. Combined with the SEM and TEM results shown in Figure 2 and Figure 4, this decline can be attributed to severe aggregation of iron-containing particles and progressive pore blockage caused by excessive iron deposition. In addition, the XRD and Fe^0^ titration results indicate that excessive iron loading promoted the formation of FeAl_2_O_4_ and iron oxide phases, which further decreased the proportion of catalytically active Fe^0^ species. Therefore, the catalytic performance of the system was governed by the synergistic effects of Fe^0^ content, pore accessibility, and active-site exposure. Only when a suitable balance between active component loading and pore structure preservation was achieved could superior catalytic performance be obtained.

#### 2.2.2. Effect of Different Supports

To further investigate the influence of support pore structure on catalytic performance, alumina supports with different specific surface areas and pore volumes were employed to prepare comparative catalysts. As shown in Table 3 and Figure 6, the degradation efficiency of RhB increased with increasing specific surface area and pore volume of the support. This result indicates that a large pore volume and high specific surface area are highly favorable for improving heterogeneous Fenton catalytic performance. Large specific surface area favors Fe^0^ dispersion, and interconnected pore channels promote mass transfer of RhB, H_2_O_2_, and reactive species. Consequently, the utilization efficiency of catalytic active sites is significantly enhanced.

In addition, adsorption experiments using the bare alumina support were conducted under identical conditions without H_2_O_2_ addition. The degradation efficiency of RhB by the support alone was only 0.52%, which was substantially lower than that achieved by the Fe^0^-loaded catalysts. This result demonstrates that adsorption alone cannot effectively remove RhB under the present conditions, whereas the introduction of Fe^0^ through carbothermal reduction significantly enhanced catalytic degradation performance.

Based on the combined analysis of XRD, SEM, TEM, and Fe^0^ titration results, C0.4-Fe0.6-Al0.5 exhibited the most favorable balance between Fe^0^ content and pore structure preservation, resulting in the highest catalytic degradation efficiency toward RhB. Therefore, this sample was selected as the representative catalyst for subsequent investigations.

#### 2.2.3. Effect of Initial Concentration

The concentration of organic pollutants in practical dye wastewater can vary over a wide range. Therefore, evaluating catalytic performance under different initial pollutant concentrations is important for assessing the potential applicability of the catalyst. The effects of initial RhB concentration on catalytic degradation performance were investigated under the conditions of catalyst dosage = 0.2 g·L^−1^, pH = 3.58, and H_2_O_2_ concentration = 10 mM.

**Figure 7:**
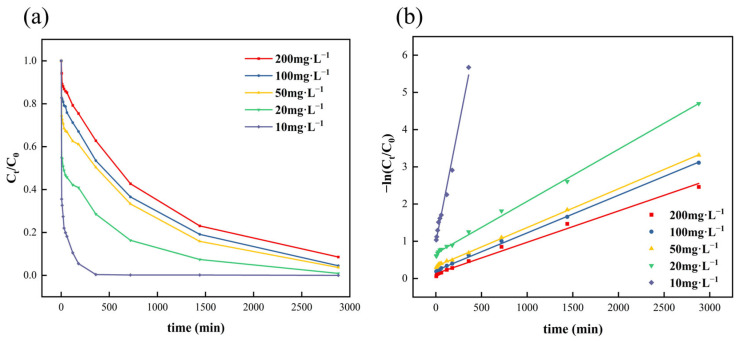
(**a**) The catalytic degradation capacity of RhB under different initial concentrations; (**b**) pseudo-first-order degradation kinetics fitting curve under different initial concentration.

As shown in Figure 7 and Table 4, the degradation efficiency gradually decreased with increasing initial RhB concentration. The highest rate (k = 0.0125 min^−1^) is achieved at 10 mg·L^−1^. This superior performance can be attributed to the large pore volume and high specific surface area of the catalyst. Under low-concentration conditions, RhB molecules can be rapidly enriched near the catalyst surface and effectively oxidized by reactive oxygen species generated during the heterogeneous Fenton reaction. The pseudo-first-order kinetic fitting results presented in Table 5 show that the apparent rate constant k decreased continuously with increasing RhB concentration. This phenomenon can be attributed to the limited number of active sites and reactive oxygen species available in the system. At high concentrations, excessive RhB molecules and intermediates compete for limited active sites and reactive oxygen species, lowering oxidation efficiency [36].

Nevertheless, the catalyst still shows satisfactory removal for high-concentration wastewater, indicating its potential applicability for the treatment of high-concentration dye wastewater.

#### 2.2.4. Effect of Solution pH

**Figure 8:**
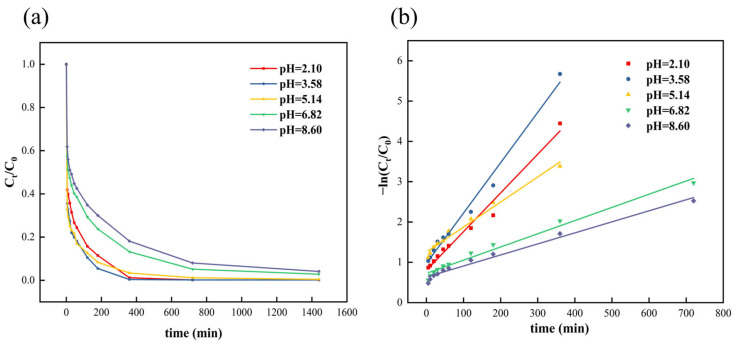
(**a**) The catalytic degradation capacity of RhB under different pH values; (**b**) the fitting curve of the pseudo-first-order degradation kinetics of RhB under different pH values.

Solution pH is one of the most critical parameters affecting heterogeneous Fenton reactions because it directly influences iron speciation, H_2_O_2_ activation efficiency, and the generation of reactive oxygen species. Therefore, the effect of initial solution pH on the catalytic degradation performance of RhB was systematically investigated. The catalytic degradation behavior of RhB at different pH values is presented in Figure 8, and the corresponding pseudo-first-order kinetic fitting results are summarized in Table 6. The experiments were conducted under the conditions of catalyst dosage = 0.2 g·L^−1^, H_2_O_2_ concentration = 10 mM, and initial RhB concentration = 200 mg·L^−1^.

As shown in Figure 8, the catalytic degradation efficiency of RhB was strongly dependent on solution pH. When the pH increased from 2.10 to 8.60, the degradation efficiency gradually decreased. The catalyst exhibited excellent degradation performance within the acidic pH range of 2.10–5.14, where the degradation efficiency after 1440 min remained close to 99.8%. In contrast, under near-neutral and alkaline conditions, the degradation efficiency decreased significantly.

The pseudo-first-order kinetic fitting results further demonstrate that the optimal pH is 3.58 (k = 0.0125 min^−1^), where the Fe^0^/Fe^2+^/Fe^3+^ redox cycle is most efficient, thereby promoting H_2_O_2_ activation and enhancing the generation of reactive oxygen species such as •OH [28]. Under neutral or alkaline conditions, iron hydrolysis and precipitation inhibit the Fenton reaction. Strong acidity (pH = 2.10) slightly reduces activity, possibly due to RhB molecular stability. Guo et al. reported that the UV-Vis absorption spectra of RhB remained relatively stable within the pH range of 3–11 [37], indicating that RhB maintained structural stability under moderately acidic conditions. Therefore, pH = 3.58 was selected as the optimal condition for subsequent experiments.

#### 2.2.5. Effect of Catalyst Dosage

**Figure 9:**
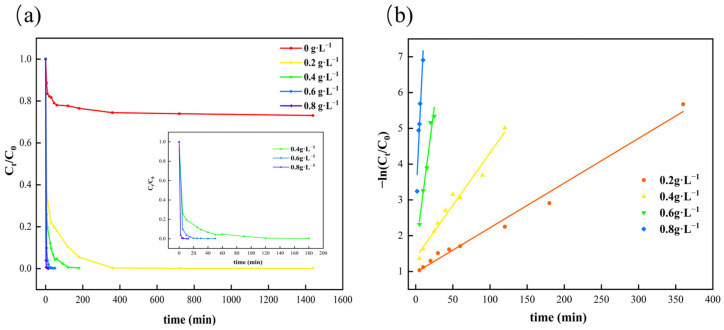
(**a**) The catalytic degradation capacity of RhB under different catalyst dosages; (**b**) pseudo-first-order degradation kinetics fitting curves of RhB under different catalyst dosages (pH = 3.58, H_2_O_2_ concentration = 10 mM, initial RhB concentration 10 mg·L^−1^).

**Figure 10:**
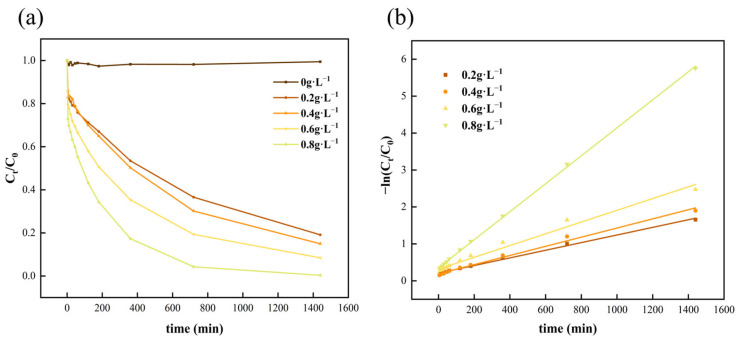
(**a**) The catalytic degradation capacity of RhB under different catalyst dosages; (**b**) pseudo-first-order degradation kinetics fitting curves of RhB under different catalyst dosages (pH = 3.58, H_2_O_2_ concentration = 10 mM, initial RhB concentration 100 mg·L^−1^).

In heterogeneous Fenton systems, catalyst dosage directly affects the number of exposed active sites, the generation efficiency of reactive oxygen species, and the contact probability between pollutants and catalytic active centers. Therefore, optimizing catalyst dosage is important not only for improving degradation efficiency but also for reducing operational cost and enhancing catalyst utilization efficiency. The effect of catalyst dosage on RhB degradation was investigated using RhB solutions with initial concentrations of 10 and 100 mg·L^−1^. The initial pH and H_2_O_2_ concentration were fixed at 3.58 and 10 mM, respectively. The catalytic degradation performance and pseudo-first-order kinetic fitting curves are shown in Figure 9 and Figure 10, while the corresponding kinetic parameters are summarized in Table 7 and Table 8.

For both low- and high-concentration RhB systems, the degradation efficiency and apparent reaction rate increased significantly with increasing catalyst dosage. This enhancement can be attributed to the increased number of exposed Fe^0^ active sites available for H_2_O_2_ activation, which promoted the generation of reactive oxygen species and accelerated RhB degradation.

For the RhB solution with an initial concentration of 10 mg·L^−1^, the apparent rate constant k increased dramatically from 0.0125 min^−1^ at a catalyst dosage of 0.2 g·L^−1^ to 0.432 min^−1^ at 0.8 g·L^−1^. A similar trend was also observed for the RhB solution with an initial concentration of 100 mg·L^−1^, although the overall degradation rate remained lower because of the increased pollutant loading and higher consumption of reactive oxygen species. Compared with the degradation rate constants of HPS-0.15LFO (photo-Fenton) [30] and K-Fe (photo-Fenton) [31] reported in Table 1, the present system exhibits faster degradation efficiency under similar reaction conditions.

Table 6 and Table 7 illustrate that as the dosage increases, the k value increases rapidly, and the degradation reaction rate is significantly enhanced. Under the condition of 0 g·L^−1^, no catalyst was added to the system; so, no degradation kinetic fitting was performed. Overall, the above data suggest that the degradation efficiency of RhB increased significantly with increasing catalyst dosage. The rate constant reaches 0.432 min^−1^ at 0.8 g·L^−1^, superior to most reported Fenton catalysts. Considering efficiency and cost, 0.6 g·L^−1^ is chosen as the optimal dosage.

#### 2.2.6. Effect of H_2_O_2_ Dosage

**Figure 11:**
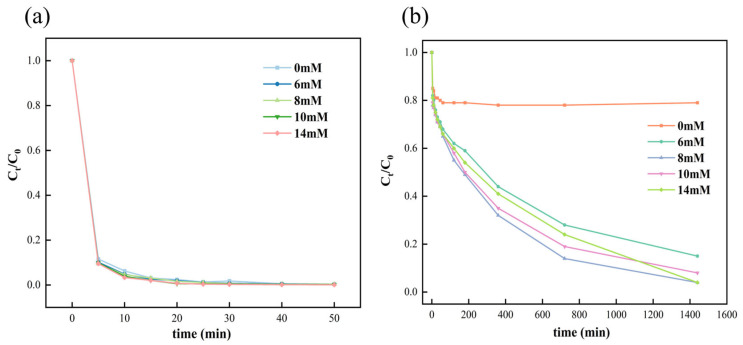
(**a**) The catalytic degradation capacity of RhB under different H_2_O_2_ dosages at an initial concentration of 10 mg·L^−1^; (**b**) at an initial concentration of 100 mg·L^−1^.

**Figure 12:**
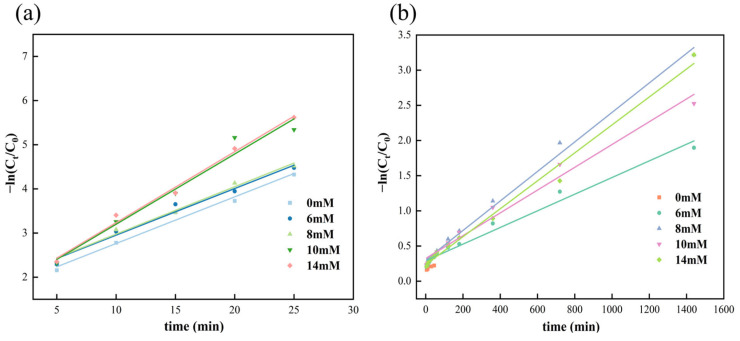
(**a**) Pseudo-first-order degradation kinetics fitting curves of RhB under different H_2_O_2_ dosages at an initial concentration of 10 mg·L^−1^; (**b**) at an initial concentration of 100 mg·L^−1^.

The effect of H_2_O_2_ dosage on the degradation of RhB dye was investigated. The dosage of C0.4-Fe0.6-Al0.5 was 0.6 g·L^−1^, pH = 3.58, and the initial concentrations of the RhB solutions were 10 and 100 mg·L^−1^. The corresponding degradation performance and pseudo-first-order kinetic fitting results are shown in Figure 11 and Figure 12, respectively. For the low-concentration RhB system (10 mg·L^−1^), the catalyst still exhibited relatively high removal efficiency even without H_2_O_2_ addition. This result indicates that adsorption enrichment contributed significantly to pollutant removal under low-concentration conditions. In contrast, for the high-concentration RhB system (100 mg·L^−1^), the degradation efficiency was relatively limited in the absence of H_2_O_2_, indicating that adsorption alone was insufficient for effective pollutant removal.

When the H_2_O_2_ dosage reached 8 mM, the degradation efficiency of RhB after 1440 min reached 96.4%, representing the optimal catalytic performance for the high-concentration system. However, further increasing the H_2_O_2_ dosage to 10 and 14 mM did not significantly improve degradation performance. Excess H_2_O_2_ causes radical self-quenching, thus reducing oxidation efficiency [28]. The pseudo-first-order kinetic fitting results shown in Table 9 and Table 10 further confirm this trend. For the low-concentration RhB system, the decolorization of the RhB solution is driven by the combined effects of adsorption enrichment and catalytic oxidation. In contrast, for the high-concentration system, when H_2_O_2_ is in excess, radical side reactions can compromise the overall degradation performance.

The above results show that after loading the active component onto Al_2_O_3_ via carbothermal reduction, the RhB degradation performance is greatly improved compared to that of Al_2_O_3_ even without adding H_2_O_2_, which may be attributed to the adsorption effect of Fe^0^ on the support surface [38]. For the high-concentration system, an H_2_O_2_ dosage of 8 mM provided the best balance between degradation performance and cost effectiveness; for the low-concentration system, due to the significant contribution of adsorption, the effect of different H_2_O_2_ dosages on the overall removal efficiency is relatively limited.

### 2.3. Reaction Mechanism 

#### 2.3.1. Radical Quenching Experiment Results

**Figure 13:**
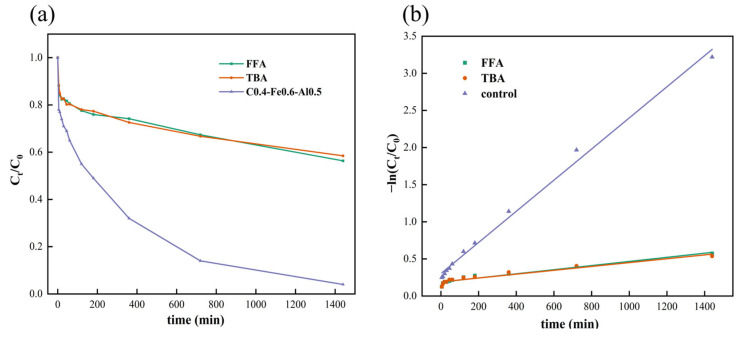
(**a**) The catalytic degradation capacity of RhB during the free radical quenching experiment; (**b**) the pseudo-first-order degradation kinetics fitting curve of RhB during the free radical quenching experiment.

To clarify the dominant reactive species involved in the heterogeneous Fenton degradation process, radical quenching experiments were conducted using tert-butanol (TBA) and furfuryl alcohol (FFA) as scavengers for •OH and ^1^O_2_, respectively. TBA is widely recognized as an efficient hydroxyl radical scavenger, whereas FFA exhibits high selectivity toward singlet oxygen [39]. The experiments were carried out under the conditions of catalyst dosage = 0.6 g·L^−1^, initial RhB concentration = 100 mg·L^−1^, pH = 3.58, and H_2_O_2_ concentration = 8 mM. The corresponding degradation performance and kinetic fitting results are shown in Figure 13 and Table 11.

After the addition of TBA and FFA, the degradation efficiency and the apparent rate constant (k) decreased markedly (Figure 13 and Table 11), demonstrating the involvement of reactive oxygen species in the catalytic degradation process [28]. The stronger inhibition caused by TBA indicates that •OH was the dominant reactive species. In contrast, the inhibitory effect of FFA suggests a possible contribution of ^1^O_2_ to RhB degradation. Nevertheless, because FFA may also react with other reactive oxygen species, additional evidence would be required to unequivocally verify the role of ^1^O_2_ in the present system.

#### 2.3.2. Reusability Experiment Results

**Figure 14:**
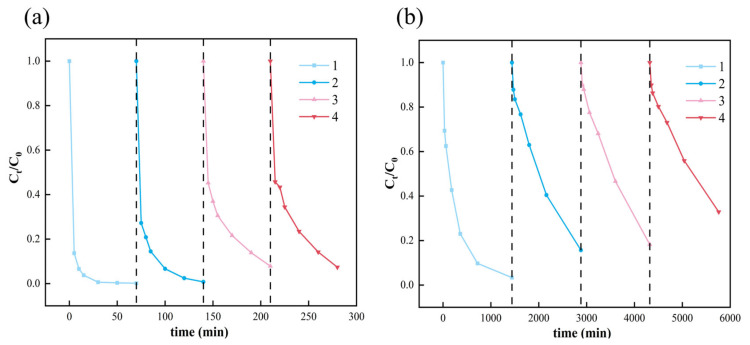
(**a**) The reuse experiment of RhB at an initial concentration of 10 mg·L^−1^; (**b**) at an initial concentration of 100 mg·L^−1^.

The cycling stability and reusability of heterogeneous Fenton catalysts are important factors affecting their practical applicability. Therefore, the reusability performance of C0.4-Fe0.6-Al0.5 was evaluated under repeated catalytic degradation conditions. As shown in Figure 14, the degradation efficiency gradually decreased with increasing cycle number for both low- and high-concentration RhB systems. For 10 mg·L^−1^ RhB, removal remains 92.6% after four cycles. In contrast, for the high-concentration RhB system (100 mg·L^−1^), the degradation efficiency decreased more significantly from 96.7% to 67.1% after four cycles.

The gradual decline in catalytic activity can be attributed to several factors. First, Fe^0^ is susceptible to oxidation during repeated exposure to aqueous solution, dissolved oxygen, and ethanol washing, resulting in the transformation of active Fe^0^ into less active high-valence iron species. Second, RhB molecules and degradation intermediates can accumulate on the catalyst surface and within the pore channels during repeated reactions. These adsorbed species may block active sites and hinder the generation of reactive oxygen species. In addition, partial iron leaching during catalytic reactions may further reduce the number of accessible active sites, thereby contributing to catalyst deactivation. The more severe performance attenuation observed in the high-concentration RhB system suggests that the accumulation of degradation intermediates and active-site blockage became more pronounced under high pollutant loading conditions.

Nevertheless, the catalyst still maintained relatively high degradation efficiency after multiple cycles, particularly in the low-concentration RhB system. This result indicates that the catalyst possesses acceptable structural stability and reusability, which can be mainly attributed to the favorable pore structure and dispersion of Fe^0^ active species within the alumina framework.

### 2.4. Degradation Pathway Analysis

To further elucidate the degradation mechanism of RhB in the heterogeneous Fenton system, the reaction solution after catalytic degradation was analyzed by liquid chromatography–mass spectrometry (LC-MS) and total organic carbon was measured by a total organic carbon analyzer. The analysis was conducted under the conditions of catalyst dosage 0.8 g·L^−1^, H_2_O_2_ concentration 8 mM, initial RhB concentration 200 mg·L^−1^, and pH = 3.58.

**Figure 15:**
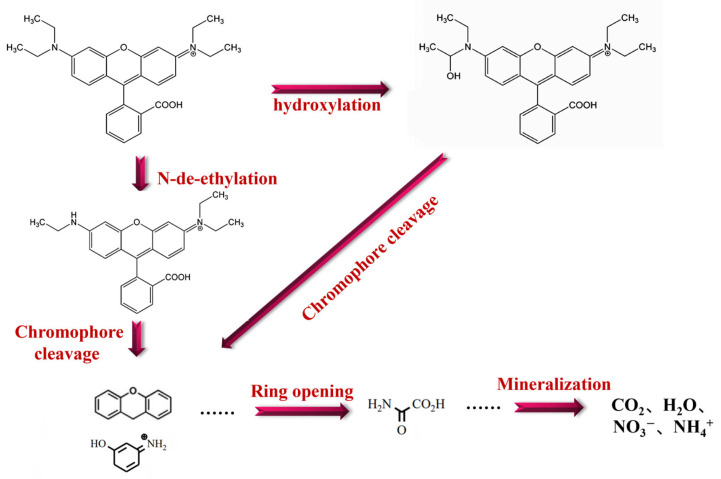
The possible pathways of RhB degradation. The arrows indicate the proposed transformation pathways, the red labels represent the different degradation reactions (including hydroxylation, N-de-ethylation, chromophore cleavage, ring opening, and mineralization), and the ellipsis (...) indicates intermediate products that are omitted for simplicity.

**Figure 16:**
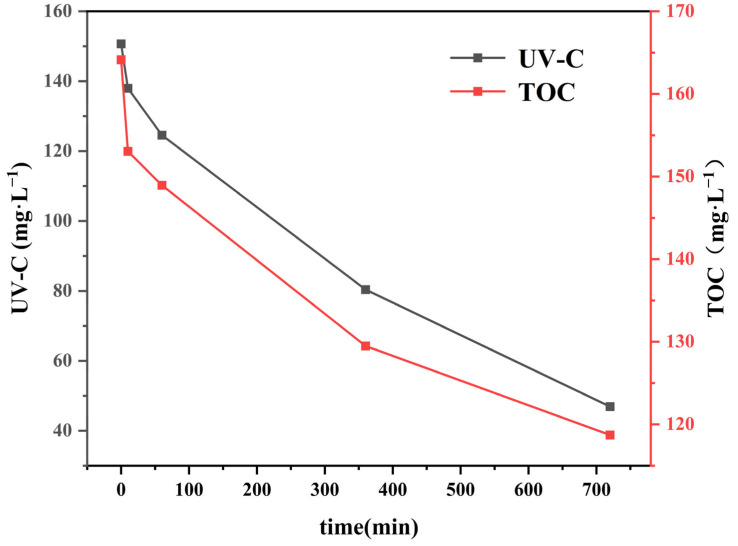
Comparison of carbon contents determined by UV-Vis spectrophotometry and TOC analysis (UV-C represents the carbon content calculated from ultraviolet measurements).

Figure 15 shows the UPLC-QTOF-MS spectrum of the RhB solution after degradation by C0.4-Fe0.6-Al0.5/H_2_O_2_. Among these, the peak at m/z 443.23 is the base peak, assignable to RhB, and the peak at m/z 444.23 corresponds to its natural isotopic peak. In addition to the parent ion, two prominent characteristic peaks at m/z 415.19 and 459.22 were also detected. Based on the detected m/z values and previously reported RhB degradation pathways [25,36,40], a possible degradation mechanism of RhB was proposed. Figure 16 shows that the carbon content gradually decreases as the degradation time increases.

As summarized in Table 12 and Figure 15, RhB degradation mainly proceeded through two parallel initial pathways. The first route follows the N-deethylation pathway, RhB lost 28 mass units to form an intermediate at m/z 415.19, corresponding to the successive removal of N-ethyl groups from the amino substituents [41]. The second pathway is initiated by the attack of hydroxyl radicals on the central carbon and xanthene chromophore of RhB, triggering hydroxylation and yielding an intermediate with m/z 459.22 [42]. In these pathways, •OH preferentially attacks the diethylamino side chains of the RhB molecule, leading to the stepwise N-deethylation of RhB into the corresponding intermediates, which are then further rapidly degraded into small molecules in subsequent reactions. Combined with Figure 16 and the chromatographic analysis, it can also be seen that RhB is not completely mineralized; instead, it is decomposed into small-molecule carbon species, which become colorless and are also easier to remove in subsequent treatment. Meanwhile, •OH can also directly add to the central carbon or the conjugated chromophoric groups of RhB, generating hydroxylated products such as C_28_H_31_N_2_O_3_^+^ and further inducing the cleavage of the chromophoric groups. As the catalytic reaction proceeded, these primary intermediates underwent further oxidation reactions, including deamination, decarboxylation, aromatic ring opening, and fragmentation into smaller oxygen-containing molecules. Eventually, the intermediates were gradually mineralized into CO_2_, H_2_O, NO_3_^−^, and NH_4_^+^ [40].

Combined with the radical quenching results, it can be concluded that •OH played a dominant role in the initial cleavage and oxidation of RhB molecules, whereas ^1^O_2_ likely participated in the subsequent oxidation of intermediate products. In addition, the large pore volume and high specific surface area of the alumina support facilitated the diffusion and enrichment of RhB molecules within the catalyst framework, thereby improving the contact efficiency among Fe^0^, H_2_O_2_, and organic pollutants. The continuous Fe^0^/Fe^2+^/Fe^3+^ redox cycle further promoted H_2_O_2_ activation and sustained reactive oxygen species generation, ultimately contributing to the efficient degradation and mineralization of RhB in the heterogeneous Fenton system.

## 3. Materials and Methods

### 3.1. Materials

**Figure 17:**
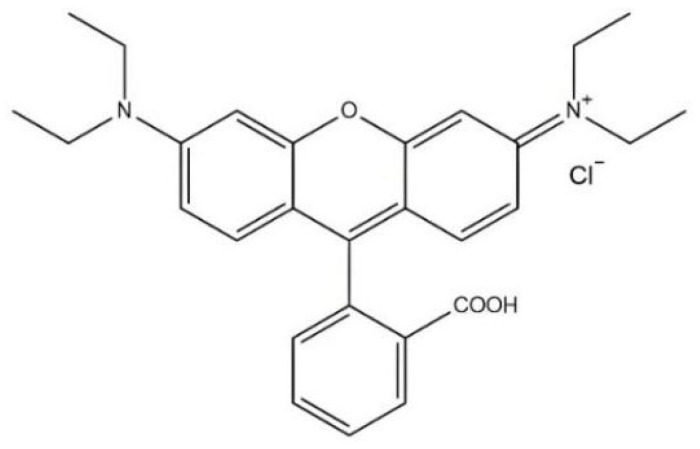
Rhodamine B molecular structure.

Rhodamine B (CR), whose molecular structure is shown in Figure 17, was purchased from Aladdin Reagent Co., Ltd. (Shanghai, China). Glucose (AR) was obtained from Tianjin Chemical Reagent Co., Ltd. (Tianjin, China), and iron(III) chloride hexahydrate (AR) was purchased from Sinopharm Chemical Reagent Co., Ltd. (Shanghai, China).Alumina was synthesized via a hydrothermal method reported previously [43].

### 3.2. Preparation of Materials

Glucose was employed as the carbon source and FeCl_3_·6H_2_O was used as the iron precursor. The molar ratios of glucose to FeCl_3_·6H_2_O were 0.75, 0.85, 1.0, 1.2, and 1.5, respectively. The support alumina was first mixed with glucose and then dried. The obtained precursor was subsequently semi-carbonized at 500 °C with a heating rate of 5 °C·min^−1^ and held for 2 h under a nitrogen atmosphere. Subsequently, FeCl_3_·6H_2_O was dissolved in deionized water and impregnated onto the semi-carbonized precursor. The mixture was ultrasonically dispersed for 20 min, stirred for 1 h, and dried at 80 °C for 12 h. The semi-carbonized precursor was placed in a quartz boat and introduced into a tubular furnace. After purging with N_2_ (100 mL·min^−1^) for 30 min, the sample was heated to 800 °C at 5 °C·min^−1^ and held for 3 h. After reduction, the sample was cooled naturally to room temperature under the same N_2_ atmosphere.

To investigate the influence of the pore structure parameters of the support on the catalytic performance, alumina samples with different proportions of pore-expanding agent added were used as supports. Referring to the preparation process of C0.4-FeZ-Al0.5, Z was fixed at 0.6. The zero-valent iron/alumina catalysts prepared using modified alumina were named C0.4-Fe0.6-Al0.5-compare1 and C0.4-Fe0.6-Al0.5-compare2, respectively.

### 3.3. Characterization of Materials

The crystal structure of the materials was characterized by X-ray diffraction (XRD, Ultima-III, Rigaku Corporation, Tokyo, Japan) using Cu Kα radiation. Before measurement, the voltage and current were set to 40 kV and 40 mA, respectively. The scanning 2θ range was 10–80° at a scan rate of 10°·min^−1^, and the patterns were matched with standard PDF cards. The morphology and particle size were examined by scanning electron microscopy (SEM, Supra 55, Zeiss, Oberkochen, Germany) and transmission electron microscopy (TEM, JEM-2100, JEOL, Tokyo, Japan). Low-temperature nitrogen adsorption-desorption measurements were tested on a TriStar II 3020 analyzer (Micromeritics, Norcross, GA, USA). Samples were degassed at 300 °C for 8 h under vacuum before measurement. The sample was cooled to room temperature, weighed, and then transferred to the analysis station. The measurement was conducted at 77 K. The pore volume was determined from the relative pressure (p/p_0_), the specific surface area was calculated using the Brunauer–Emmett–Teller (BET) equation, and the pore size distribution was analyzed by the Barrett–Joyner–Halenda (BJH) method from the adsorption branch of the isotherm. The content of zero-valent iron was determined according to the Chinese standard GB/T 6730.6-2016 [44]. The degradation products of RhB were identified using ultra-performance liquid chromatography coupled with quadrupole time-of-flight mass spectrometry (UPLC-QTOF-MS, Waters, Milford, MA, USA). Chromatographic separation was performed on a C18 column using water containing 0.1% formic acid (mobile phase A) and acetonitrile containing 0.1% formic acid (mobile phase B) as the eluents. The flow rate was maintained at 0.3 mL·min^−1^, the column temperature was set at 35 °C, and the injection volume was 10 μL. Mass spectrometric analysis was carried out with an electrospray ionization (ESI) source operated in positive ion mode, and spectra were recorded over an m/z range of 50–800 to elucidate the degradation pathway of RhB. Total organic carbon (TOC) was determined using a Shimadzu TOC-L CPH analyzer (Kyoto, Japan).

### 3.4. RhB Catalytic Degradation Experiment

Catalytic degradation of RhB over catalysts with different iron loadings: Catalytic degradation of RhB was carried out in a 100 mL solution at room temperature with magnetic stirring at 600 r·min^−1^. After adding the catalyst, the suspension was stirred for 30 min to reach adsorption–desorption equilibrium, and then H_2_O_2_ was added to initiate the reaction. After sampling and filtration of the reaction solution, the solution was diluted to an appropriate concentration range if necessary. The absorbance was then measured at a wavelength of 554 nm, and the concentration was calculated using the standard curve. The degradation performance of RhB was evaluated. The degradation performance was expressed as *C_t_*/*C*_0_, and the degradation efficiency D was calculated using Equation (2):

(2) $$D\;=\;(C_{0}\;-\;C_{t})/C_{0}\;\times \;100\%$$

where C_0_ (mg/L) is the initial concentration of RhB, and C_t_ (mg/L) is the concentration of RhB at reaction time t.

Degradation kinetics experiment: The degradation kinetics were fitted and evaluated using the pseudo-first-order kinetic model [45], and calculated by Equation (3):

(3) $$-ln\frac{C_{t}}{C_{0}}\;=\;kt$$

where k (min^−1^) is the pseudo-first-order degradation rate constant.

Experiment on the effect of support specific surface area and pore volume on catalyst performance: 20 mg of C0.4-Fe0.6-Al0.5-compare1 and 20 mg of C0.4-Fe0.6-Al0.5-compare2 were separately added into 100 mL of RhB solution with an initial pH of 3.58 and an initial concentration of 200 mg·L^−1^. A magnetic stir bar was placed into the mixture, which was then stirred on a magnetic stirrer at 600 r·min^−1^. Then, 10 mM H_2_O_2_ was added, and the reaction was allowed to proceed for 6 h at room temperature. The degradation efficiency D was measured. The effects of initial concentration, solution pH, catalyst dosage, and H_2_O_2_ dosage on the catalytic degradation of RhB were further investigated.

### 3.5. Radical Quenching Experiment

To identify the main reactive species in the reaction system, C0.4-Fe0.6-Al0.5 was selected as the representative sample. 60 mg of the catalyst was added to 100 mL of RhB solution with an initial concentration of 100 mg·L^−1^ and pH 3.58, followed by the addition of 8 mM H_2_O_2_. Subsequently, tert-butanol (TBA, 200 mM) and furfuryl alcohol (FFA, 20 mM) were respectively added. After reacting for different time intervals under stirring, the supernatant was sampled, filtered, and the absorbance was measured at 554 nm [39].

### 3.6. Reusability Experiment

Under the conditions of 60 mg catalyst, pH 3.58, and initial RhB concentrations of 10 mg·L^−1^ and 100 mg·L^−1^ (each in 100 mL solution), 8 mM H_2_O_2_ was introduced while stirring magnetically at 600 r·min^−1^. After each reaction cycle, the catalyst was separated, and the supernatant was sampled, filtered, and the absorbance of RhB was measured at a wavelength of 554 nm. The separated catalyst was washed with ethanol, recovered by centrifugation, and then dried at 80 °C for 6 h for use in the next cycle of reusability experiments.

## 4. Conclusions

A zero-valent iron/alumina heterogeneous Fenton catalyst was successfully synthesized via a glucose-assisted carbothermal reduction method, and its catalytic performance toward Rhodamine B (RhB) degradation was systematically investigated. Combined characterization results from XRD, SEM, TEM, and Fe^0^ titration confirmed the successful generation and uniform dispersion of Fe^0^ on the alumina support. The catalytic activity strongly depended on the balance between Fe^0^ loading and preservation of the pore structure. Excessive iron loading resulted in severe pore blockage and aggregation of iron species, whereas the catalyst with moderate iron loading exhibited the best catalytic performance. The catalyst showed excellent RhB degradation efficiency under acidic conditions. Under the optimal reaction conditions (pH = 3.58, catalyst dosage = 0.8 g·L^−1^, and H_2_O_2_ concentration = 10 mM), complete degradation of RhB (10 mg·L^−1^) was achieved within 25 min, with an apparent pseudo-first-order rate constant of 0.432 min^−1^. Radical quenching experiments indicated that hydroxyl radicals (•OH) were the dominant reactive species responsible for RhB degradation, while singlet oxygen (^1^O_2_) also contributed to the oxidation process. UPLC-QTOF-MS analysis revealed that RhB degradation mainly proceeded through N-deethylation and hydroxylation pathways, followed by chromophore cleavage, aromatic ring opening, and eventual conversion into smaller intermediates and inorganic products. Despite partial deactivation caused by Fe^0^ oxidation, iron leaching, and blockage of active sites, the catalyst maintained satisfactory reusability and structural stability. These findings demonstrate that designing alumina supports with large pore volumes and high specific surface areas is an effective strategy for improving Fe^0^ dispersion, enhancing interfacial mass transfer, and increasing the accessibility of active sites in heterogeneous Fenton systems. This study provides valuable insights into the rational design of high-performance iron-based heterogeneous Fenton catalysts for dye wastewater treatment.

## Figures and Tables

**Table 2:** BET surface area and pore volume of Fe^0^/Al_2_O_3_ catalysts prepared using different alumina supports and FeCl_3_ loadings.

Sample	SA(m^2^·g^−1^)	PV(cm^3^·g^−1^)	Da (nm)
Al_2_O_3_	540.8	2.24	16.5
Al_2_O_3_-compare1	364.7	0.77	8.44
Al_2_O_3_-compare2	456.1	1.68	14.7
C0.4-Fe0.4-Al0.5	208.1	0.28	5.38
C0.4-Fe0.6-Al0.5	136.2	0.22	6.46
C0.4-Fe0.8-Al0.5	105.3	0.20	7.59

SA—specific surface area, PV—pore volume, Da—average pore diameter.

**Table 3:** The catalytic degradation efficiency of RhB by catalysts with different ferric chloride impregnation amounts.

Catalysts	C_t_/C_0_	D%
C0.4-Fe0.4-Al0.5	0.613	38.6
C0.4-Fe0.5-Al0.5	0.595	40.4
C0.4-Fe0.6-Al0.5	0.583	41.7
C0.4-Fe0.7-Al0.5	0.745	25.5
C0.4-Fe0.8-Al0.5	0.793	20.7
C0.4-Fe0.6-Al0.5-compare1	0.683	31.7
C0.4-Fe0.6-Al0.5-compare2	0.616	38.4

**Table 4:** Degradation efficiency (D) of RhB under different initial concentrations.

C_0_ (mg·L^−1^)	10	20	50	100	200
t (min)					
5	64.5	45.2	25.5	17.2	5.8
10	67.4	45.8	27.1	17.9	10.9
20	72.6	49.3	29.2	19.1	11.9
30	77.9	51.2	31.5	20.8	13.1
45	80.1	53.4	32.7	21.3	14.1
60	81.8	54.3	33.1	24.1	14.8
120	89.5	57.8	37.5	28.8	20.8
180	94.5	59.1	38.9	32.9	24.6
360	99.7	71.5	49.7	46.5	37.2
720	99.8	83.7	66.7	63.4	57.3
1440	99.9	92.6	84.2	80.9	77.0
2880	100	99.1	96.4	95.5	91.5

**Table 5:** Fitting of pseudo-first-order degradation kinetics for RhB under different initial concentrations.

C_0_ (mg·L^−1^)	10	20	50	100	200
Kinetic Constant					
k (min^−1^)	0.0125	0.00142	0.00104	0.00102	0.000841

**Table 6:** Fitting of pseudo-first-order degradation kinetics for RhB under different pH values.

pH	2.10	3.58	5.14	6.82	8.60
Kinetic Constant					
k (min^−1^)	0.00965	0.0125	0.00622	0.00327	0.00274

**Table 7:** Fitting of pseudo-first-order degradation kinetics for RhB under different catalyst dosage conditions (pH = 3.58, H_2_O_2_ concentration = 10 mM, initial RhB concentration 10 mg·L^−1^).

Dosage (g·L^−1^)	0.2	0.4	0.6	0.8
Kinetic Constant				
k (min^−1^)	0.0125	0.0293	0.159	0.432

**Table 8:** Fitting of pseudo-first-order degradation kinetics for RhB under different catalyst dosages (pH = 3.58, H_2_O_2_ concentration = 10 mM, initial RhB concentration 100 mg·L^−1^).

Dosage (g·L^−1^)	0.2	0.4	0.6	0.8
Kinetic Constant				
k (min^−1^)	0.00102	0.00124	0.00160	0.00379

**Table 9:** The fitting of the pseudo-first-order degradation kinetics of RhB under different H_2_O_2_ dosages (pH = 3.58, catalyst dosage 0.6 g·L^−1^, initial RhB concentration 10 mg·L^−1^).

Dosage of H_2_O_2_ (mM)	0	6	8	10	14
Kinetic Constant					
k (min^−1^)	0.106	0.106	0.107	0.159	0.161

**Table 10:** The fitting of the pseudo-first-order degradation kinetics of RhB under different H_2_O_2_ dosages (pH = 3.58, catalyst dosage 0.6 g·L^−1^, initial RhB concentration 100 mg·L^−1^).

Dosage of H_2_O_2_ (mM)	0	6	8	10	14
Kinetic Constant					
k (min^−1^)	0.00154	0.00118	0.00216	0.00160	0.00199

**Table 11:** Fitting of pseudo-first-order degradation kinetics for the radical quenching experiment.

Quenching Agent	FFA	TBA	Control
Kinetic Constant			
k (min^−1^)	0.000278	0.000259	0.00216

**Table 12:** The degradation products during the catalytic degradation of RhB by C0.4-Fe0.6-Al0.5/H_2_O_2_.

m/z	Molecular Formula	Proposed Structure
443.2263	C_28_H_31_N_2_O_3_^+^	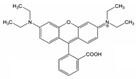
415.1935	C_26_H_27_N_2_O_3_^+^	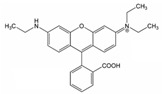
459.2158	C_28_H_31_N_2_O_4_^+^	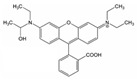
89~182	-	-

## Data Availability

The original contributions presented in this study are included in this article. Further inquiries can be directed to the corresponding author.
